# Assessment of preoperative axillary nodal disease burden: breast MRI in locally advanced breast cancer before, during and after neoadjuvant endocrine therapy

**DOI:** 10.1186/s12885-022-09813-9

**Published:** 2022-06-25

**Authors:** Joana Reis, Joao Boavida, Hang T. Tran, Marianne Lyngra, Laurens Cornelus Reitsma, Hossein Schandiz, Woldegabriel A. Melles, Kjell-Inge Gjesdal, Jürgen Geisler, Jonn Terje Geitung

**Affiliations:** 1 Department of Diagnostic Imaging and Intervention, Akershus University Hospital (AHUS), Postboks 1000, 1478 Lørenskog, Norway; 2 Institute of Clinical Medicine, Campus AHUS, University of Oslo, Postboks 1000, 1478 Lørenskog, Norway; 3 Translational Cancer Research Group, Akershus University Hospital (AHUS), Postboks 1000, 1478 Lørenskog, Norway; 4 Department of Pathology, Akershus University Hospital (AHUS), Postboks 1000, 1478 Lørenskog, Norway; 5 Department of Breast and Endocrine Surgery, Akershus University Hospital (AHUS), Postboks 1000, 1478 Lørenskog, Norway; 6 Sunnmøre MR-Clinic, Agrinorbygget, Langelansveg 15, 6010 Ålesund, Norway; 7 Department of Oncology, Akershus University Hospital (AHUS), Postboks 1000, 1478 Lørenskog, Norway

**Keywords:** Locally Advanced Breast Cancer, Neoadjuvant, Endocrine Therapy, MRI, Lymph Nodes, Metastases

## Abstract

**Background:**

Axillary lymph node (LN) metastasis is one of the most important predictors of recurrence and survival in breast cancer, and accurate assessment of LN involvement is crucial. Determining extent of residual disease is key for surgical planning after neoadjuvant therapy. The aim of the study was to evaluate the diagnostic reliability of MRI for nodal disease in locally advanced breast cancer patients treated with neoadjuvant endocrine therapy (NET).

**Methods:**

Thirty-three clinically node-positive locally advanced breast cancer patients who underwent NET and surgery were prospectively enrolled. Two radiologists reviewed the axillary nodes at 3 separate time points MRI examinations at baseline (before the first treatment regimen), interim (following at least 2 months after the first cycle and prior to crossing-over), and preoperative (after the final administration of therapy and immediately before surgery). According to LN status after surgery, imaging features and diagnostic performance were analyzed.

**Results:**

All 33 patients had a target LN reduction, the greatest treatment benefit from week 8 to week 16.

There was a positive correlation between the maximal diameter of the most suspicious LN measured by MRI and pathology during and after NET, being highest at therapy completion (*r* = 0.6, *P* ≤ .001). Mean and median differences of maximal diameter of the most suspicious LN were higher with MRI than with pathology. Seven of 33 patients demonstrated normal posttreatment MRI nodal status (yrN0). Of these 7 yrN0, 3 exhibited no metastasis on final pathology (ypN0), 2 ypN1 and 2 ypN2. Reciprocally, MRI diagnosed 3 cases of ypN0 as yrN + . Diffusion -weighted imaging (DWI) was the only axillary node characteristic significant when associated with pathological node status (χ^2^(4) = 8.118, *P* = .072).

**Conclusion:**

Performance characteristics of MRI were not completely sufficient to preclude surgical axillary staging. To our knowledge, this is the first study on MRI LN assessment following NET in locally advanced breast cancer, and further studies with larger sample sizes are required to consolidate the results of this preliminary study.

**Trial Registration:**

Institutional Review Board approval was obtained (this current manuscript is from a prospective, open-label, randomized single-center cohort substudy of the NEOLETEXE trial). NEOLETEXE, a phase 2 clinical trial, was registered on March 23^rd^, 2015 in the National trial database of Norway and approved by the Regional Ethical Committee of the South-Eastern Health Region in Norway; registration number: REK-SØ-84–2015.

**Supplementary Information:**

The online version contains supplementary material available at 10.1186/s12885-022-09813-9.

## Background

In breast cancer, the presence and extent of axillary LN metastasis are one of the most important predictors of overall recurrence and survival, and precise assessment of LN involvement is a crucial component in axillary staging [1, 2]. While the 5-year survival rate for patients with disease localized to the breast is 98.8%, the figure declines to 85.8% for patients with metastatic regional LN [2]. The nodal status often determines the need for systemic therapy, surgical treatment, and radiation therapy [3].

NET is emerging as a very effective strategy in the treatment of “large T2” or locally advanced breast cancers, optimizing surgical outcomes, improving survival, and reducing recurrences [4–7]. NET has become a useful approach to luminal-like tumors with strongly hormone receptor positive (HR +) expression, mainly in postmenopausal or/and elderly women. Pathological complete response (pCR) is rare with NET, clinical response rates do result in increased eligibility for breast conserving therapy (BCT) by decreasing tumor size and by reducing axillary nodal disease burden, hence diminishing the rates of axillary LN dissection (ALND) procedures [6, 7]. Over the past years, the surgical treatment regimen for axillary LN metastases assessment has evolved from routine ALND toward less extensive procedures, such as the sentinel LN biopsy (SLNB) [1, 8–10]. Previous studies have proven SLNB to be a safe technique when cN1 axilla changed to ycN0 after NAC [11–13]. Most authors have concluded that NET is less likely to diminish surgery in the axilla than in the breast, even though pCR rates range from 0% to 13.3% [7, 14, 15]. However, SLNB remains associated with morbidity, such as seroma, hematoma, lymphedema, neuropathy, and pain [16]. To overcome this, a noninvasive axillary nodal staging technique that could substitute SLNB, precisely determine LN-negative breast cancer patients and consequently prevent SLNB associated morbidity would therefore be important.

Breast MRI is routinely performed for monitoring response to NET [7, 17, 18]. MRI permits radiologists to simultaneously evaluate breast tumor and axillary LNs in the same field of view. Additionally, breast MRI can expose internal mammary and supraclavicular LN involvement and provide additional prognostic information for extensive nodal disease. Previous studies reported that the MRI sensitivity and specificity of axillary imaging are 57–72% and 54–72%, respectively, and accuracy ranging from 60–87% after NAC [8, 19, 20]. Alternative noninvasive imaging techniques, such as ultrasound or PET-CT, have been used. Although MRI scans do not use ionizing radiation (compared to PET-CT or CT), and exhibit lower intra- and interobserver variability (as in ultrasound examinations) [21, 22].

To the best of our knowledge, MRI findings associated with residual disease by sequential monitoring of axillary response to NET- before, during and after- have not been investigated. In this article, we hypothesize that breast MRI can be used to acquire information on LN morphology to determine axillary staging. Axillary response monitoring during NET could be beneficial to directly observe therapeutic efficacy and proper duration, and to a better selection and personalization of the treatment, i.e., monitoring responders and LN-negative breast cancer patients to a more patient-tailored treatment strategy reducing systemic overtreatment; and in poor- or non-responders to change inefficient treatment, switch to neoadjuvant chemotherapy (NAC) and/or expedite surgery; as well as the possibility to evaluate any biological or molecular changes that may lead us to explore new biomarkers, whereas there is no measurable disease to observe when systemic therapy is given in the adjuvant setting [7, 15, 21–23]. The present study aims to evaluate the diagnostic reliability of MRI and correlation with pathology (gold standard) for axillary nodal assessment in locally advanced breast cancer patients treated neoadjuvant with endocrine therapy.

## Methods

This current manuscript is from a prospective, open-label, randomized single-center cohort substudy of the NEOLETEXE trial. NEOLETEXE, a phase 2 clinical trial, was registered on 23/03/2015 in the National trial database of Norway and approved by the Regional Ethical Committee of the South-Eastern Health Region in Norway (registration number: REK-SØ-84–2015, https://rekportalen.no) [24]. This substudy was performed in accordance with Helsinki declaration, and written informed consent was obtained from all participants [24].

### Study population

**Fig. 1:**
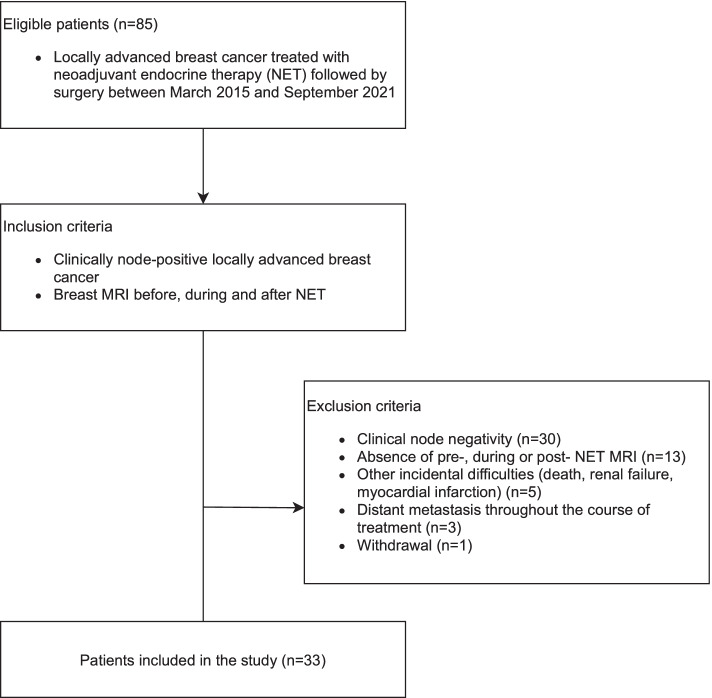
Flowchart of the study population

We prospectively enrolled 85 consecutive patients with histologically confirmed unilateral HR + and human epidermal growth factor receptor-2 negative (HER-2) locally advanced breast cancer treated with NET followed by surgery between March 2015, and September 2021. All participants had to be postmenopausal to benefit from NET with no or limited distant metastases. Based on the clinical history and radiologic findings, we excluded patients with clinically node negativity (*n* = 30), absence of MRI before, during or after NET (*n* = 13), with history of other incidental difficulties (*n* = 5), distant metastasis throughout the course of treatment (*n* = 3), and we had only one withdrawal from the study (Fig. 1). The inclusion and exclusion criteria are given in Supplementary Table 1. Finally, 33 patients who demonstrated clinically node-positivity by ultrasound and/or MRI and underwent MRI at 3 different time points were enrolled in this study (Fig. 1). The clinical node status before the first treatment regimen (i.e., at baseline) was defined with radiological findings with or without fine-needle aspiration biopsy or core needle biopsy. These radiological findings considered suggestive of metastasis included cortical thickening greater than 3 mm, fatty hilum loss or displacement, the presence of eccentric cortical thickening, and irregular margin, principally when distinctly different from contralateral axillary LNs. Of them, representative LNs of 23 patients were histologically confirmed malignant. Patient selection for neoadjuvant treatment was determined by the multidisciplinary breast cancer team at the Akershus University Hospital and the NET regimen was based on the current NEOLETEXE trial protocol [24]. The NET intra-patient cross- over regimen consisted of one of the following treatment arms: (1.) letrozole 2.5 mg o.d. for at least 8 weeks thereafter continuing with exemestane 25 mg o.d. for at least another 8 weeks prior to surgery; and (2.) exemestane 25 mg o.d. for at least 8 weeks thereafter continuing with letrozole 2.5 mg o.d. for at least another 8 weeks prior to surgery [18, 24, 25].

### Imaging technique

Breast MRI was performed at baseline (prior treatment) as well as after at least 8 weeks after the first cycle, and after approximately 16 weeks (immediately prior surgery) on treatment with a Philips Ingenia 1.5-T MRI unit (Philips Healthcare, Best, Netherlands) by using a dedicated 16-channel bilateral breast coil with parallel imaging capabilities with the patient in supine position and both arms elevated with close contact between coil and axillae. The imaging protocol consisted of the following: prior to the administration of contrast of an axial turbo spin echo (TSE) T1-weighted sequence, an axial single-shot echo planar (SS-EPI) DWI with three respective *b* values (0,50, 800), and a three-dimensional T2-weighted with fat suppression [17, 18]. Two dynamic sequences were then applied in an interleaved pattern prior and during the injection of the contrast agent. The high temporal resolution images were acquired using a 3D TI_T2* weighted multi-echo planar imaging (EPI) sequence and intercalated with a dynamic high spatial resolution three-dimensional T1-weighted turbo field echo (TFE) sequence. Both contrast- enhanced imaging sequences include a total scanning time of approximately 7 min with a full coverage of both breasts with no slice gap. Details on MRI sequences are described on Supplementary Table 2.

### Image analysis and interpretation

Images from all MRI scans were prospectively analyzed by consensus of two radiologists (J.B and J.R) who were blinded to the clinical-pathologic information. The following imaging characteristics were evaluated by MRI: number of suspicious nodes, short axis diameter of the most suspicious node, presence of perinodal infiltration, cortical thickness, shape, hilum and abnormal restricted diffusion. The axillary LNs were considered suspicious for metastasis when at least one of the following findings were noted by ultrasound and/or MRI at baseline (first time point), and only by MRI following at least 2 months after the first cycle and prior to crossing-over (second time point), and after the final administration of therapy and immediately before surgery (third time point): cortical thickness greater than 3 mm, the presence of eccentric cortical thickening, irregular margin, and/or fatty hilum loss or displacement [26, 27].

### Histopathologic analysis, sentinel node mapping and axillary surgery

After NET all patients underwent BCT or mastectomy, and axillary LNs were surgically removed by SLNB, ALND or both. If residual axillary LN metastasis was suspected following physical or MRI examination, then the patient underwent ALND. For all other patients, SLNB was performed. If no metastasis was found on the frozen sentinel LN, then no further ALND was performed. However, if pathologic examination of the frozen sections for sampled nodes revealed metastases, ALND was performed [26, 28].

### Data collection and statistical analysis

The clinical-pathologic data collected included age at cancer diagnosis; pretreatment clinical TN stage (cTN); histologic type; histologic grade; type of tumor and axillary surgery; and posttreatment pathologic TN stage (ypTN), according to the standard ypTN (8^th^. edition) restaging system of the largest contiguous focus of invasive cancer (T stage) and the extent of regional LN involvement (N stage); y indicates that patients had received neoadjuvant treatment (Table 1) [29, 30]. ypN0 was defined as the complete absence of metastases.

**Table 1:** Patient demographics and tumor characteristics

Characteristic	Value
No. of patients	33
Age (y)	
Mean^a^	74.4 ± 6.7
Range	58- 84
Lymphadenopathy at US	
Negative	4
Positive	29
Clinical T stage	
T2	2 (6.1)
T3	11 (33.3)
T4	20 (60.6)
Clinical N stage	
N0	14 (42.4)
N1	16 (48.5)
N2	2 (6.1)
N3	1 (3.0)
Clinical stage	
IIB	4 (12.12)
IIIA	7 (21.21)
IIIB	20 (60.61)
IIIC	1 (3.03)
IV	1 (3.03)
Histologic type	
Ductal	26 (78.8)
Lobular	5 (15.2)
Other	2 (6.1)
Histologic grade	
1	1 (3.0)
2	26 (78.8)
3	5 (15.2)
missing	1 (3.0)
Breast Surgery	
Breast-conserving surgery	4 (87.9)
Total mastectomy	29 (12.1)
Lymph node surgery	
SLNB	7 (21.21)
ALND	18 (54.55)
Both	8 (24.24)
ypT stage	
Tis	1 (3.0)
T1	8 (24.2)
T2	19 (57.6)
T3	3 (9.1)
T4	2 (6.1)
ypN stage	
N0	6 (18.2)
N1	17 (51.5)
N2	9 (27.3)
N3	1 (3.0)

Unless specified otherwise, data are number of cases, with percentages in parentheses

*ALND* Axillary Lymph Node Dissection, *SLNB* Sentinel Lymph Node Biopsy, *US* Ultrasound

^a^ Data are means ± standard deviations

Descriptive statistics were used to compare MRI measurements of maximal diameter of the most suspicious node at 3 different time points to assess pathologic outcome. The association between categorical variables at baseline (US, MRI, and fine-needle aspiration biopsy and core needle biopsy) and ypN status was studied by Cramer’s V. The effect of the difference of maximal diameters between MRI and pathological assessments was performed using ANOVA followed by the Pearson correlation coefficient (r). MRI features of axillary LNs after NET (posttreatment MRI) according to the ypN status were analyzed by the Chi-square test and Fisher’s exact test. All statistical analyses were performed by using SPSS (version 27.0). *P* ≤ 0.05 was considered to indicate a statistically significant difference.

## Results

### Patient characteristics

In total, 33 patients with clinically node-positive locally advanced breast cancer were included (mean age ± standard deviation, 74.4 years ± 6.7; range, 58–84 years). Patient and tumor characteristics are summarized in Table 1. The most common histologic type was invasive ductal carcinoma of the breast (78.8%, 26 of 33). The most clinical T and N stages of study population were cT4 stage (60.6%, 20 of 33) and cN1 stage (48.5%, 16 of 33).

From the 33 patients of the study population with clinically node-positive disease pre-NET, 29 (29/33, 87.9%) patients demonstrated radiologic node-positive by diagnostic US, and 27 (27/33, 81.8%) patients by baseline MRI. Diagnostic pathologic axillary LN metastasis was confirmed in 23 (23/33, 69.70%) patients by using fine-needle aspiration biopsy or core needle biopsy. No relationships between diagnostic US- and baseline MRI examinations with posttreatment pathology were noticed (Cramer’s V:0.066; *P* = 0.706; Cramer’s V: 0.185; *P* = 0.287, respectively). Nevertheless, the liaison between fine-needle aspiration biopsies or core needle biopsies with posttreatment pathology was significantly different and moderate (Cramer’s V:0.617; *P* = 0.002).

Among the 33 patients, 4 (12.1%) underwent BCT and 29 (87.9%) underwent total mastectomy after therapy. The LN surgery method was SLNB in 7 (21.21%), ALND in 18 (54.55%) and both in 8 (24.24%).

### Assessments outcomes and response evaluation

Based on the means from ANOVA, statistically significant differences were revealed among the MRI measurements at the 3 different time points and pathology (Huynh–Feldt: 898.030, *F* (3, 46.582) = 15.209, *P* < 0.001). Based on MRI assessment of the maximal diameter of the most suspicious LN, the initial mean was 14.3 mm, after approximately 8 weeks of treatment, the mean was 12.7 mm, and following completion of 16 weeks of intended treatment, mean was 10.0 mm, compared to pathological mean 7.5 mm. At baseline MRI, the median LN size as measured by the sum of maximal diameters of target LN for all patients was 13.0 mm (range, 0.0–34.0). At interim MRI, the median was 13.0 mm (range, 0.0–31.0), and at posttreatment MRI, it was 9.0 mm (range, 0.0–25.0). Of the 33 patients who completed treatment and underwent surgery, pathological median was 7.0 mm (range, 0.0–25.0). Overall, patients had a target LN reduction, with the greatest treatment benefit from week 8 to week 16.

**Fig. 2:**
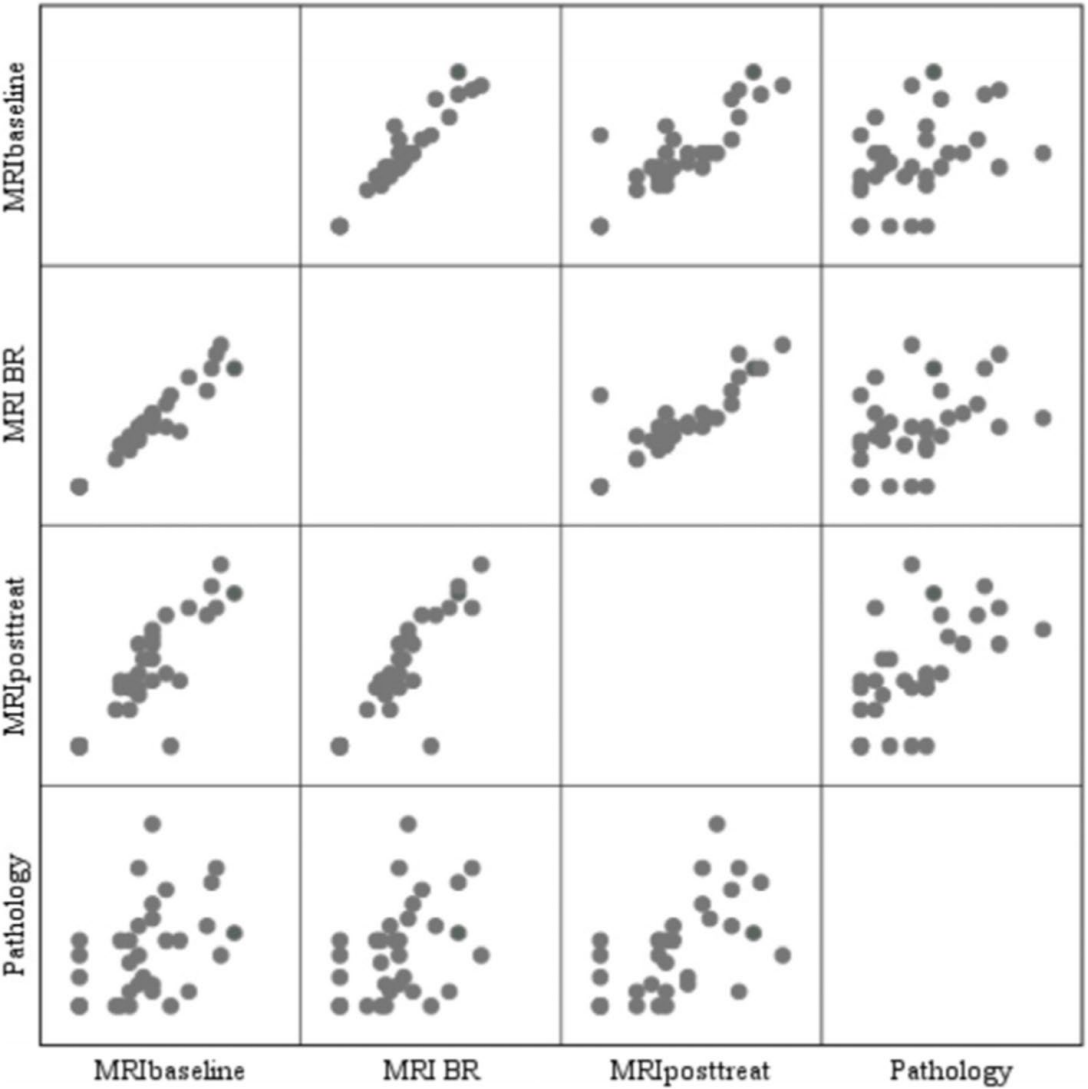
Matric Scatter plots illustrate the correlation between the difference of maximal diameters between MRI and pathological assessments. MRI scans at baseline, between regimens and posttreatment. The correlation between posttreatment MRI lymph node size and pathology was positive, moderate and higher (*r* = 0.6, *P* ≤ .001) compared to the correlation between MRI at baseline and between regimens (*r* = 0.431, *P* ≤ .05; *r* = 0.425, *P* ≤ .05, respectively). Among all the three MRI examinations, the correlation coefficient was highest and very high positive between MRI at baseline and between regimens (*r* = 0.97, *P* ≤ .001). BR: between regimens, NET: neoadjuvant endocrine therapy; Posttreat: posttreatment; r: the Pearson correlation coefficient

When the maximal diameter of the most suspicious LN obtained from all of the 3 MRI examinations were correlated with the pathological maximal diameter of the most suspicious LN, all correlations were positive. The correlation between posttreatment MRI LN size and pathology was positive, moderate and higher (*r* = 0.6, *P* ≤ 0.001) compared to the correlation between MRI at baseline and between regimens (*r* = 0.431, *P* ≤ 0.05; *r* = 0.425, *P* ≤ 0.05, respectively). Among all the 3 MRI examinations, the correlation coefficient was highest between MRI at baseline and between regimens (*r* = 0.97, *P* ≤ 0.001) (Fig. 2). Posttreatment MRI nodal status (yrN) was normal in 7 of 33 patients, i.e., yrN0 (21.21%), of which 3 underwent SLNB, 1 went directly to ALND and 3 performed both SLNB and ALND. Of these 7 yrN0, 3 had negative nodes (ypN0) and 4 had nodal metastasis on final pathology (2 patients showed ypN1 and 2 showed ypN2). In 26 of 33 patients (78.79%), the axillary nodes remained abnormal on posttreatment MRI (yrN +), 17 underwent directly to ALND, 4 underwent SLND and 5 performed both SLNB and ALND. Twenty-three of these 26 patients had residual disease (ypN +) and 3 exhibited no metastasis (ypN0) on final pathology. Of the 33 clinically node-positive patients (rN +), 6 were ypN0 (18.18%). However, only one (4.35%) patient of these 6 had biopsy-proven nodal disease at diagnosis.

Of the 6 ypN0, we found that 5 were invasive ductal carcinoma of the breast, and only 1 was invasive lobular carcinoma of the breast.

The most common abnormal axillary imaging feature at posttreatment MRI was the presence of abnormal restricted diffusion (18/26), followed by the loss of hilum (16/26). Only the presence of abnormal restricted diffusion showed a trend to be statistically significant with ypN status (χ^2^(4) = 8.118, *P* = 0.072). Otherwise, there was no statistically significant difference and trend among the others axillary node characteristics at posttreatment breast MRI (Table 2).

**Table 2:** Axillary node characteristics at posttreatment breast MRI associated with ypN status

MRI Parameter (33)	ypN0 (6)	ypN1-2 (26)	ypN3 (1)	*P* Value
Number of suspicious nodes				
• ≤ 3	6	21	1	.704
• 4–9	0	4	0	
• ≥ 10	0	1	0	
Maximal diameter of the most suspicious node				
• < 10 mm	6	13	0	.155
• 10–19 mm	0	10	1	
• ≥ 20 mm	0	3	0	
Perinodal infiltration				
• Absent	6	13	1	.412
• Present	0	13	0	
Cortical thickness				
• < 10 mm	6	18	1	.568
• 10–19 mm	0	7	0	
• ≥ 20 mm	9	1	0	
Hilum				
• Normal	3	6	1	.138
• Displaced/Loss	3	20	0	
Shape				
• Oval	4	16	1	.816
• Round	2	7	0	
• Irregular	0	3	0	
Abnormal restricted diffusion				
• Absent	4	5	1	.072
• Present	1	17	0	
• Missing	1	4	0	

Unless specified otherwise, data are number of cases

*MRI* Magnetic Resonance Imaging

## Discussion

NET is a less toxic alternative to NAC for patients with HR + tumors, but whether the effect on the axillary nodes is comparable with that of NAC is dubious [7]. Although MRI is regularly used to determine residual disease in the primary breast tumor, there are limited data in assessing axillary nodal staging and estimating axillary LNs metastasis. Few data are available on the ability of breast MRI to evaluate axillary LN status either prior to start of therapy or after completion of systemic therapy [2, 16, 19–21, 26, 27]. The significance of nodal response with NET is inconclusive, since pCR is infrequent after NET of conventional duration [31]. According to Geisler et al., when NET is administered for 3–4 months (independently of the drug used), pCR is a rare event [32]. Thus, NET is now given for 6 months at least, in general. Consequently, the rate of therapeutic effect clearly increases as the NET period is extended. In our sample population a period of 4–6 months of NET with an intra-patient cross-over regimen using exemestane and letrozole was relatively effective. There was a positive correlation between the maximal diameter of the most suspicious LN measured by MRI and pathology during and after NET, being strongest at completion of therapy.

Previous studies reported that axilla pCR after NET ranged from 0 to 13.3% [7]. In this study, we found that 4.35%% of all biopsy-proven clinically node-positive patients becoming ypN0 after NET. Weiss et al. has also found similar higher rates (17%) of pCR [7, 33]. However, the overall ypN0 of 4.35% in our study is in accordance with the expected rates [7, 14, 16, 19, 20, 31, 33].

Of the 6 ypN0, only 1 was invasive lobular carcinoma of the breast. This is consistent with the recent study by Thornton et al. who compared data to determine the outcome of invasive lobular carcinomas treated with NAC and NET [14]. As claimed by Montagna et al., in the neoadjuvant setting, NAC may be more successful than NET in lobular cancer. Nevertheless, the shorter duration of NET may contribute to these findings.

**Fig. 3.**
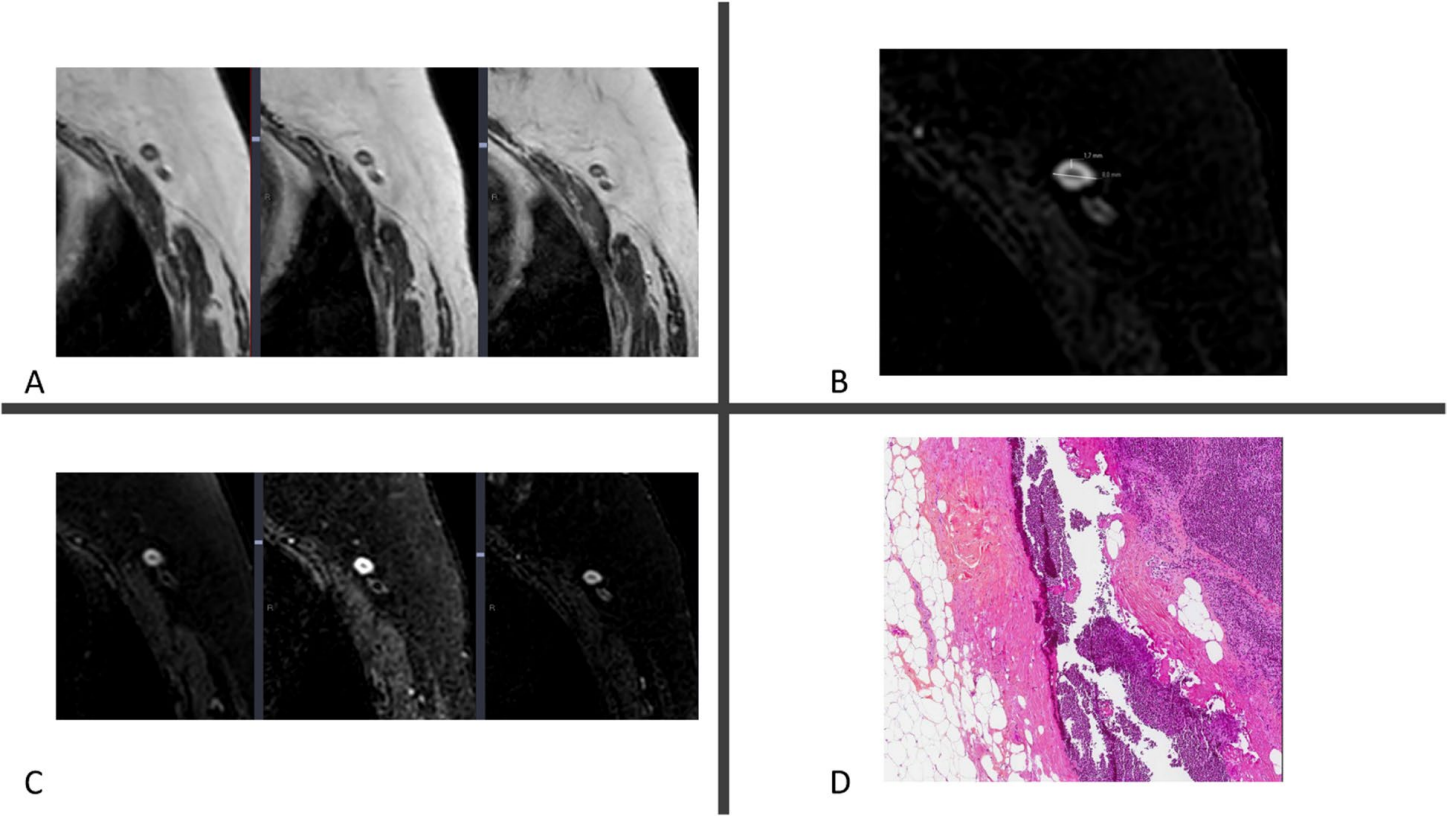
Sixty-nine year-old woman with ER + /HER2- locally advanced breast cancer on the left side treated with NET protocol, mastectomy and ALND. **A**, **B** and **C**, Axial T2-weighted (**A**) and T1-weighted (**B** and **C**) MR images show LNs with normal appearances at all MRI scans (from left to right, **A** and **C**: baseline, interim and posttreatment) throughout the course of treatment. **B** T1-weighted MR image shows a normal oval LN with cortical thickning of 1.7 mm and maximal diameter of 8 mm. **D** Corresponding photomicrograph (H and E, original magnification × 200) shows normal nodal tissue

**Fig. 4.**
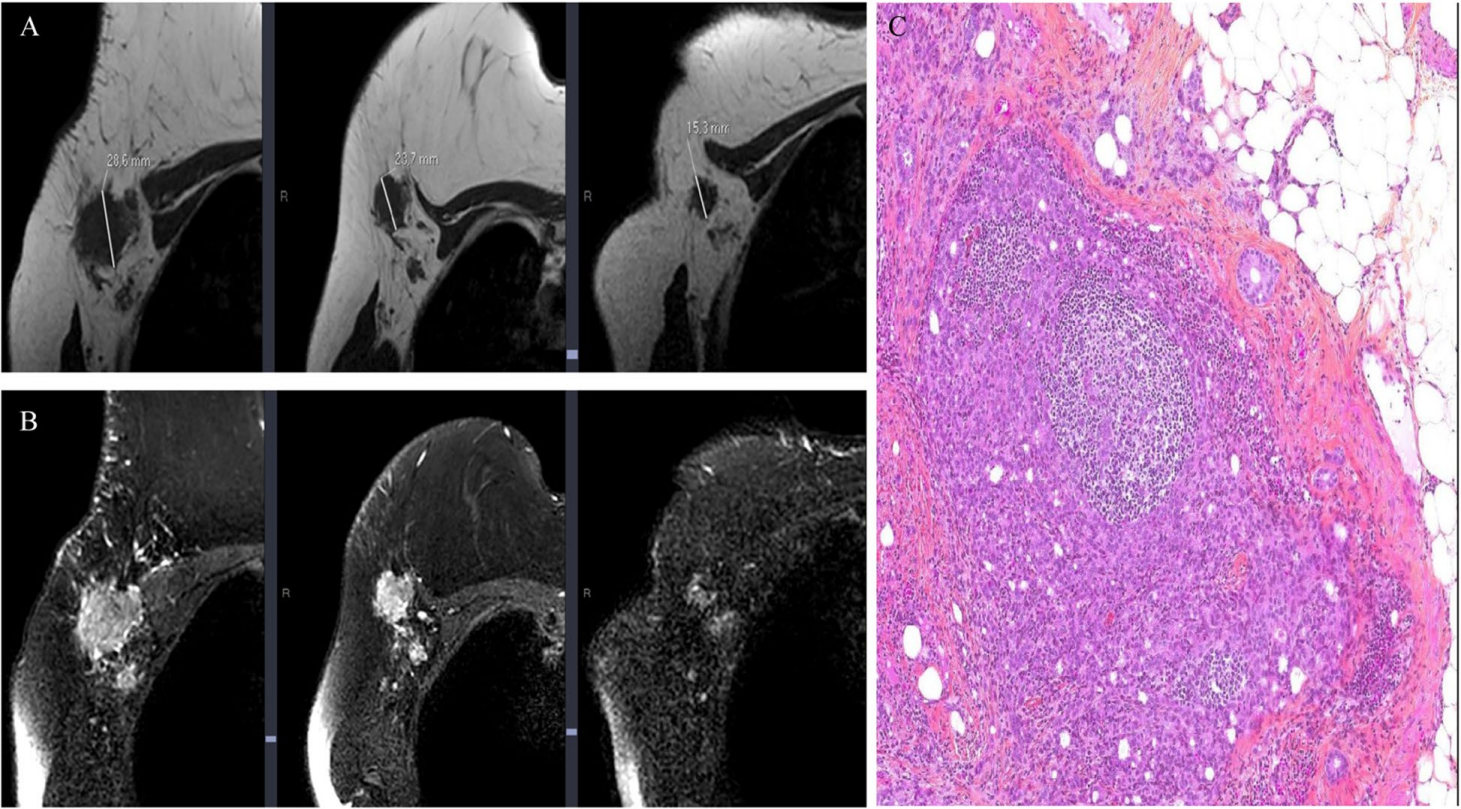
Fifty-Eight year-old woman with ER + /HER2- locally advanced breast cancer on the right side treated with NET protocol, mastectomy and ALND. **A** and **B** Axial T2-weighted and T1-weighted MR images, respectively. From left to right: baseline T2-weighted (**A**) and T1-weighted (**B**) show a metastatic LN with irregular edge, displaced hilum, signal inhomogeneity, perinodal infiltration and maximal diameter of 28.6 mm; images obtained during and after NET, T2-weighted (**A**) and T1-weighted (**B**), shows that the node continues to have irregular edge, displaced hilum, that signal inhomogeneity and perinodal infiltration persist, and maximal diameter of 23.7 mm and 15.3 mm, respectively, these findings indicate malignancy. **C** Corresponding photomicrograph (H and E, original magnification × 200) shows widespread tumor deposition in LN with irregular border at baseline

In contrast to the definition of breast imaging complete response, which is the absence of any enhancement, the definition of imaging complete response in the LNs is difficult to determine due to normal variations in architecture and size among patients, besides the lack of ability to use enhancement as criteria for axillary LNs malignancy (benign LNs normally enhance) [20, 22]. Standard breast MRI is limited in LN assessment: exact cortical measurement and morphologic nodal change from round to oval on MRI may change depending on patient position, respiratory motion artifact from the chest wall, and potential false identification of LNs from adjacent vascular structures [34]. In our data analysis, MRI LN size overestimated compared to pathology. Furthermore, LNs are rated as suspicious if the short axis diameter is greater than 1 cm (or a long-to-short axis ratio of less than 2). Cortical thickening, loss of fatty hilum, round shape, irregular margin, inhomogeneous cortex, perifocal edema, and asymmetry of LNs in terms of number or size compared with the contralateral side are additional typical morphological findings suggestive of metastasis (Figs. 3 and 4) [20, 34]. In our observations, 4 cases were discordantly diagnosed as yrN0 but were ypN2/N1. Reciprocally, we found that MRI diagnosed 3 cases of ypN0 as yrN + . The overlap in size between metastatic, hyperplastic and normal LNs and the fact that micrometastases in small LNs are common, all these findings contribute to a higher rate of false- negative and positive interpretations.

Kuijs et al. claimed that micrometastases will be difficult to detect with any imaging technique. In recent literature small metastases, such as isolated tumor cells (i.e., N0i + , < 0.2 mm) and micrometastases (i.e., N1mi, 0.2*–*2.0 mm), do not influence overall survival [20, 22]. Accordingly, the need to achieve higher sensitivity rates for detecting micrometastases is less desirable, creating an opportunity for axillary nodal staging by MRI [22].

DWI, although the only axillary node characteristic that demonstrates a trend to be statistically significant when associated with ypN status, encounters the issue of a current lack of standardization across breast centers. DWI and apparent diffusion coefficient (ADC) map could not reflect heterogeneity in diffusion within a single LN, not since only metastasis, but also reactive hyperplasia or fibrotic proliferation, might affect diffusion [16].

Our study has several strengths, including its prospective nature, the availability of our unique cohort of patients diagnosed with locally advanced breast cancer treated with NET and radiology review of MRIs at baseline, interim and posttreatment by two experienced radiologists. To our knowledge, this is the first study that investigates the use of breast MRI in the evaluation of nodal response after NET compared to pathologic assessment as gold standard.

We acknowledge certain limitations. First, this is a single-institution study with a small number of patients, preventing us from evaluating trends over time. Second, determination of clinically positive LNs by US and MRI and undergoing subsequent histological confirmation. Thus, the results may have suffered from selection bias and underestimation of clinical stage. Third, interpretation of axillary LN was based on the most suspicious finding among the axillary LNs. We did not use special marking techniques, we concluded that the LN with the most suspicious imaging finding represents the clinically suspicious LN.

Our findings confirm that despite imaging response assessment by MRI, surgical resection with pathologic evaluation of the breast and axillary LNs remains necessary to reliably validate pCR and ensure resection of microscopic residual disease. More research is warranted regarding optimizing the duration of NET and the prognostic value of axillary residual disease during and after NET, as well as increased representation of patients with locally advanced breast cancer in prospective randomized clinical trials. Emerging MRI techniques that combine functional and perfusion information such as diffusion, metabolism, and hypoxia are needed and will improve MRI accuracy. In addition, research on machine-learning techniques, radiomics, and radiogenomics with the goal of predicting response on pretreatment imaging improving outcomes and enhance care for this population are ongoing [35–37].

## Conclusions

The findings are worthy of consideration in a larger cohort of patients, as the importance of breast MRI in prediction of axillary response after NET has not been sufficiently investigated. As the trend towards less aggressive axillary surgery continues, a more accurate, yet encompassing role for imaging will be required in staging axillary disease. We believe that our study is a contribution to a continuous and important task of improving imaging in general and MRI especially in diagnostics and treatment of breast cancer.

## Supplementary Information

**Additional file 1:** **Supplementary table 1. **Inclusion and exclusion criteria. **Supplementary table 2. **Details of breast magnetic resonance sequences acquisition.

## Data Availability

The datasets generated during and analyzed during the current study are not publicly available due to the fact that the authors will use the raw data as an exclusive source for data mining to publish additional papers later but are available from the corresponding author on reasonable request.

## Abbreviations

ALND: Axillary lymph node dissection
BCT: Breast conserving therapy
DWI: Diffusion-weighted imaging
ER: Estrogen receptor
HER2: Human epidermal growth factor receptor 2
HR: Hormone receptor
LN: Lymph node
NAC: Neoadjuvant chemotherapy
NET: Neoadjuvant endocrine therapy
o.d.: Once daily
pCR: Pathological complete response
SLNB: Sentinel lymph node biopsy
