# Supplementary material for: Assessment of preoperative axillary nodal disease burden: breast MRI in locally advanced breast cancer before, during and after neoadjuvant endocrine therapy
Source: BMC Cancer. 2022 Jun 25;22:702. doi: 10.1186/s12885-022-09813-9 (PMC9233812; doi:10.1186/s12885-022-09813-9)
Supplement: Supplementary file 1 — Additional file 1: Supplementary table 1. Inclusion and exclusion criteria. Supplementary table 2. Details of breast magnetic resonance sequences acquisition. [file 12885_2022_9813_MOESM1_ESM.docx]

**Supplementary material**

Supplementary table 1. Inclusion and exclusion criteria.

| **Inclusion Criteria** | **Exclusion Criteria** |
| --- | --- |
| Postmenopausal status | Triple-negative breast cancer, HER2+ status |
| ER+/HER-2- Locally advanced breast cancer  (according to AJCC TNM 8^th^ ed) | Life-threatening metastasis at diagnosis or during treatment, clinically progressive disease |
| No or very limited distant metastasis | Previous therapy for breast cancer within the last 12 months and/or medications that may interfere with endocrine therapy |
|  | Lack of any of the MRIs, lack of sufficient imaging quality |
|  | Inconclusive pathological examination |
|  | Refusal of surgery |

AJCC: The American Joint Committee on Cancer, ER+: Estrogen receptor-positive, HER-2: Human epidermal growth factor receptor 2, MRI: magnetic resonance imaging, TNM: tumor node metastasis

Supplementary table 2. Details of breast magnetic resonance sequences acquisition.

| **Sequence** | **TR/TE (ms)** | **Flip angle** | **Dyn Reps.** | **NSA** | **FOV (mm)** | **Slice thickness (mm)** | **Acquisition time** |
| --- | --- | --- | --- | --- | --- | --- | --- |
| **Pre- contrast** |  |  |  |  |  |  |  |
| T1W- TSE | 487/8 | NA | 1 | 2 | 280x340 | 3 | 2.40 min |
| DWI SSh SE- EPI | 7000/103 | NA | 1 | 3 | 280x340 | 3 | 5.50 min |
| FS 3DT2W | 1300/145 | NA | 1 | 1 | 370x370 | 2.2 | 5.20 min |
| **After a single injection of CA** |  |  |  |  |  |  |  |
| 3DT1_T2* multi-echo EPI | 38/6.2/8.8 | 28º | 41 | 1 | 280x340 | 8 | 2.84 sec/volume |
| 3D T1W THRIVE | 5.4/2.6 | 12 º | 6 | 1 | 360x120 | 2 | 56.5 sec/volume |

CA: contrast agent, DWI SSh SE- EPI: diffusion weighted imaging, single- shot, echo planar imaging with two respective b factors (50, 800), T1W THRIVE: high spatial resolution 3D T1- weighted turbo field echo (TFE) sequence, EPI: echo planar imaging, FOV: field of view, NA: non-applicable, NSA: number of signals, FS: fat suppression, TE: time echo, TR: repetition time, TSE: turbo spin echo, T1W: T1- weighted, T2W: T2- weighted, 3D: 3 dimensional.
